# 3D Convolutional Neural Network for Predicting Clinical Outcome from Coronary Computed Tomography Angiography in Patients with Suspected Coronary Artery Disease

**DOI:** 10.1007/s10278-025-01667-4

**Published:** 2025-09-30

**Authors:** Era Stambollxhiu, Leonard Freißmuth, Lukas Jakob Moser, Rafael Adolf, Albrecht Will, Eva Hendrich, Keno Bressem, Martin Hadamitzky

**Affiliations:** 1 Department of Cardiovascular Radiology and Nuclear Medicine,, TUM University Hospital, German Heart Center, Lazarettstrasse 36, Munich, 80636 Germany; 2 Smart Robotics Lab, Department of Computer Engineering, Technical University of Munich, Munich, Germany; 3 Diagnostic and Interventional Radiology, University Hospital Zurich, University of Zurich, Zurich, Switzerland; 4 School of Medicine and Health, Technical University of Munich, Munich, Germany

**Keywords:** Convolutional neural networks, Coronary computed tomography angiography, Outcome prediction, Coronary artery disease, Risk stratification

## Abstract

This study aims to develop and assess an optimized three-dimensional convolutional neural network model (3D CNN) for predicting major cardiac events from coronary computed tomography angiography (CCTA) images in patients with suspected coronary artery disease. Patients undergoing CCTA with suspected coronary artery disease (CAD) were retrospectively included in this single-center study and split into training and test sets. The endpoint was defined as a composite of all-cause death, myocardial infarction, unstable angina, or revascularization events. Cardiovascular risk assessment relied on Morise score and the extent of CAD (eoCAD). An optimized 3D CNN mimicking the DenseNet architecture was trained on CCTA images to predict the clinical endpoints. The data was unannotated for presence of coronary plaque. A total of 5562 patients were assigned to the training group (66.4% male, median age 61.1 ± 11.2); 714 to the test group (69.3% male, 61.5 ± 11.4). Over a 7.2-year follow-up, the composite endpoint occurred in 760 training group and 83 test group patients. In the test cohort, the CNN achieved an AUC of 0.872 ± 0.020 for predicting the composite endpoint. The predictive performance improved in a stepwise manner: from an AUC of 0.652 ± 0.031 while using Morise score alone to 0.901 ± 0.016 when adding eoCAD and finally to 0.920 ± 0.015 when combining Morise score, eoCAD, and CNN (*p* < 0.001 and *p* = 0.012, respectively). Deep learning–based analysis of CCTA images improves prognostic risk stratification when combined with clinical and imaging risk factors in patients with suspected CAD.

## Introduction

Over the past decade, coronary computed tomography angiography (CCTA) has become a well-established method to identify or rule out coronary artery disease (CAD), particularly in patients with low to intermediate risk [1, 2]. In addition to anatomic depiction and grading of coronary artery stenosis, CCTA offers the advantage of characterizing atherosclerotic plaque features that hold prognostic importance for the course of the disease. Such features include low-attenuation plaque, spotty calcification, remodeling, and napkin-ring sign [3, 4]. 

Semi-quantitative scores, such as the Segment Involvement Score (SIS), are routinely employed in clinical practice for risk stratification, management, and guidance of CAD patients [5]. Despite the appeal of integrating CCTA-derived plaque assessment into daily practice, its widespread clinical use is still limited by the time-consuming process and the requirement for highly trained professionals [6].

Advances in artificial intelligence and machine learning have become increasingly important in medical research, enabling the automated extraction of complex patterns from large-scale clinical datasets [7]. Deep learning algorithms have been applied to a wide range of cardiac imaging applications, from plaque type analysis, calcium score quantification, lumen segmentation, or segmentation and volume analysis of epicardial fat tissue [6, 8–10].

Convolutional Neural Networks (CNNs), a subtype of deep learning algorithms, are largely used in image analysis. They consist of multi-layered networks that simulate some properties of the human visual cortex [11]. CNNs utilize mathematical models to pass on the results to the next layer. This enables associations to be made based on previous experience and the algorithm to be trained to increase the probability of correct classification. Different types of model architectures are employed for classification, detection, and segmentation of medical imaging data [12–14].

Existing methods for risk prediction of cardiac events often lack in accuracy, emphasizing the need for research to improve patient prognosis [15]. Due to successful application of CNNs in many medical imaging and recognition tasks, we consider whether they can help improve risk assessment and prediction of major cardiac events (MACE) [16] [17]. Annotated images of coronary arteries have been used on a 2D CNN model for risk prediction in patients undergoing CCTA. Without annotation information, this model failed to improve prediction [18]. However, manual annotation of all plaques is either extremely time consuming or needs additional specialized software. Therefore, the aim of this study is to develop an optimized 3D model eliminating the need of manual plaque annotation and capable of predicting major cardiac events from unannotated CCTA images in patients with suspected coronary artery disease.

## Materials and Methods

This retrospective, single-center study is approved by the local institutional review board of Technical University Munich (Trial Number 485/S and 20/20 S), which acts based on the Declaration of Helsinki. All enrolled patients provided written informed consent prior to examination.

### Study Population

**Fig. 1:**
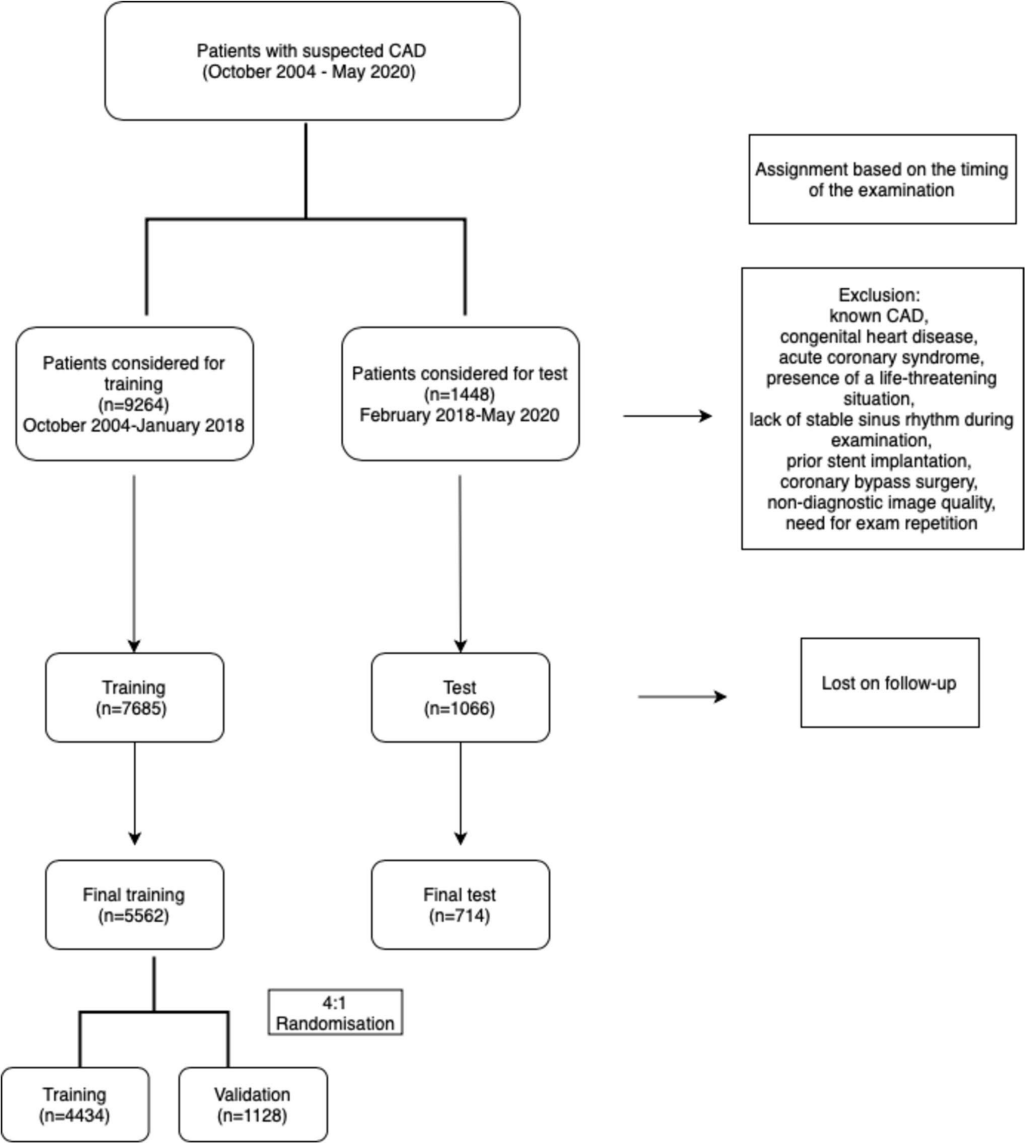
Patient enrollment flow chart

Consecutive patients above the age of 18 years undergoing CCTA for CAD at the German Heart Center Munich between October 2004 and May 2020 were enrolled in this retrospective study. The cohort was divided into two datasets based on the timing of examination: patients who underwent CCTA between October 2004 and January 2018 (*n* = 5562) were assigned to the training and validation dataset, while those examined between February 2018 and May 2020 (*n* = 714) were assigned to the test dataset. The training and validation dataset was randomly split in a 4:1 ratio, stratified by endpoint, sex, median age, and scanner generation to create the final training and validation subsets. Ultimately, 4434 and 1128 patients were assigned to these subsets, respectively (Fig. 1).

Patients with previously known CAD, congenital heart disease, acute coronary syndrome, presence of a life-threatening situation, lack of stable sinus rhythm during the examination, prior stent implantation, or coronary bypass surgery did not meet inclusion criteria. Studies with non-diagnostic image quality or the need for examination repetition were excluded from the analysis. Before examination, a structured interview was performed, including patient age, height, and weight, as well as history of cardiac disease, present concerns, and current medication.

Laboratory results and cardiac risk factors were assessed. The pre-test probability of CAD was calculated using the Morise score [19], which includes age, gender, risk factors, and symptoms to predict the probability of obstructive CAD. Follow-up information was gathered either through clinical visits, questionnaires sent by mail, or telephone contact.

### Study Endpoint

Combined endpoint of the study consists of a composite of all-cause death, myocardial infarction, unstable angina, or revascularization events.

### Image Acquisition

Throughout the study period, four different CT scan generations were used for image acquisition:

a 64-slice single source CT system from October 2004 to September 2006 (Somatom 64), a 64-slice dual source CT system from October 2006 to March 2009 (Somatom Definition), a 128-slice dual source CT system from April 2009 to May 2014 (Somatom Definition Flash), and a 192-slice dual source CT system from June 2014 to May 2020 (Somatom Force, all Siemens Healthineers). Only the last scanner generation was used for the test dataset.

Depending on the patient’s heart rate and in the absence of contraindications, intravenous beta-blocker medication (Metoprololtartrat-Omnipharm, Recordati Pharma) was administered targeting a heart rate less than 60 beats/min. Sublingual nitrates (Nitrolingual akut Spray; G. Pohl-Boskamp GmbH & Co. KG) were applied if systolic blood pressure was higher than 100 mmHg.

The coronary prospective ECG-synchronized CTA was triggered into the diastolic phase (70% of RR-interval). Tube voltage was selected by the technician and/or physician between 70 and 120 kVp, and tube current was adapted automatically based on body size (CARE Dose). Contrast circulation time was determined using a test bolus with 10 ml of contrast media (Imeron 350; Bracco Imaging GmbH), followed by a 50-ml 0.9% saline chaser (Isotonic saline solution; Fresenius Kabi GmbH). The coronary CT angiogram was performed with a 50-ml contrast bolus at 5.0 ml/s, followed by 30 ml 0.9% saline chaser.

### Clinical Plaque Assessment from CCTA

Coronary artery luminal stenosis was evaluated and interpreted by two experienced radiologists and graded as none (0%), non-obstructive (1–49%), and obstructive (≥ 50%). The most severe stenosis was considered for assessing the extent of coronary artery disease. According to presence of any plaque and the presence of coronary arteries with obstructive CAD (defined as ≥ 50% stenosis), the extent of coronary artery disease (eoCAD) was classified as normal, non-obstructive, or obstructive CAD [5].

### Image Annotation and Preprocessing

The 3D dataset was analyzed using commercially available software (Syngo.via; Siemens Healthineers) and the coronary artery tree was segmented automatically with manual correction of inconsistencies. This yielded centerlines of all three coronary arteries and the main branches.

**Fig. 2:**
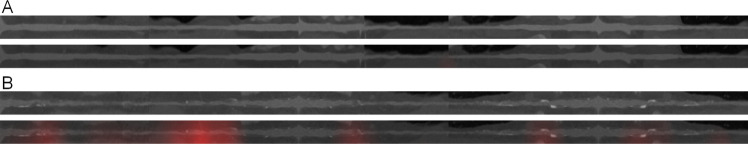
Input data without (**A**) and with (**B**) activation map

Coronary arteries were reformatted into 3D datasets consisting of multiple short-axis reformations perpendicular to the centerline. Up to five reformations (one for RCA, two for the LAD territory, and two for LCx territory) were then concatenated to a matrix of 32 × 32 × 1024 voxels with 0.6-mm spacing. If necessary, either the longest coronary branches were shortened or the matrix was filled up by zeros (Fig. 2).

#### Model Architecture and Model Training

A purpose-built 3D convolutional neural network model was used, which was inspired by DenseNet [20], whose main proposition is that densely connected layers allow for more compact models with features being reused across the network, as well as implicit deep supervision through a direct gradient flow from the loss function to each layer. Overall, this leads to a smoother loss landscape [21]. In the context of 3D CT datasets, where the number of implicit features grows cubically with input resolution as opposed to quadratic growth with 2D images, we deem efficient feature reuse and good gradient flow paramount for meaningful training and inference.

**Fig. 3:**
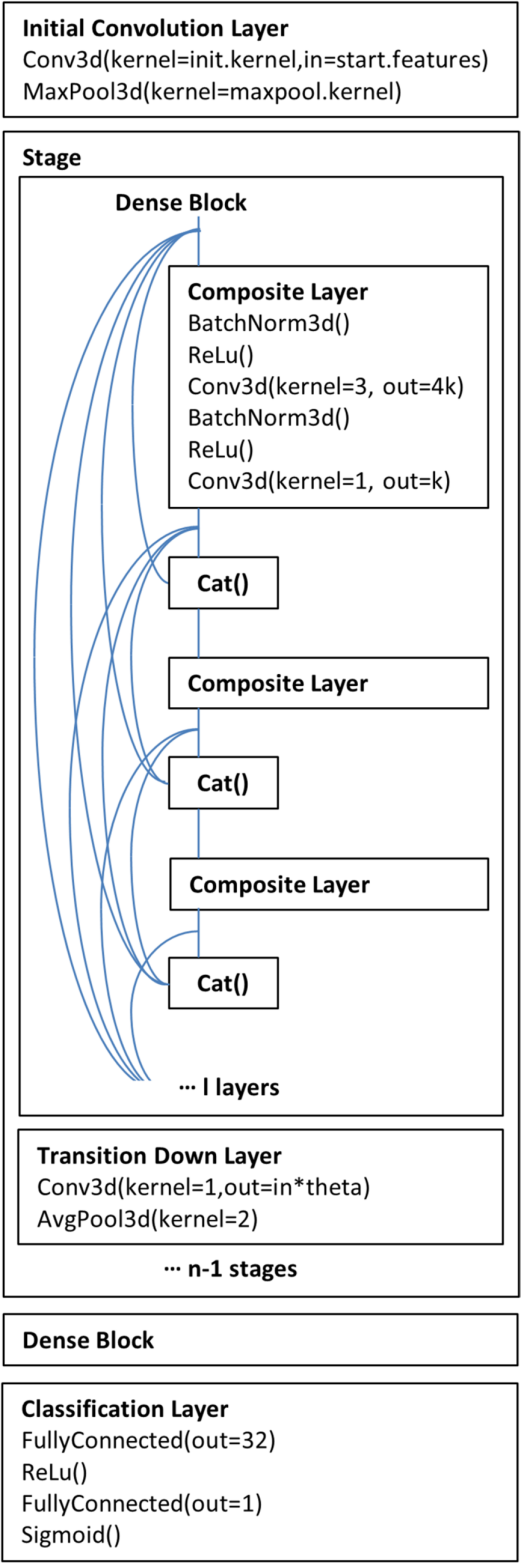
Overview of the model architecture

Our model (Fig. 3) follows the architecture of DenseNet decomposing the pipeline into dense blocks separated by transition layers.

Inside a dense block, composite layers consisting of three-dimensional convolution and batch normalization layers as well as ReLU activations project the input into a *k*-dimensional feature space. The concatenation of all previous composite layers’ outputs serves as the input for the next composite layer.

This concatenation would lead to computationally infeasible feature dimensions, so after l layer, a transition layer reduces feature dimensionality via convolutions with kernel size 1 and feature resolution using average pooling with kernel size 2.

The overall model comprises *n* pairs of dense blocks and transition layers, the exact number being part of the hyperparameter tuning, followed by a final dense block and a classification layer which, using fully connected layers, ReLU, and sigmoid layers, projects the features into the one-dimensional end point probability.

Activation maps were generated showing the absolute weights of the last convolution layer before global averaging.

The model was initialized with random weights and extensive hyperparameter search was performed in a grid search pattern to find the parameters reported in Table 1. Model training was performed in Python 3.11.5 (open-source; Python Software Foundation) by using PyTorch 1.7.1 and scikit-learn 0.23.2 libraries on a GPU workstation with an 8-core Intel Core i7 9700 K CPU at 3.6 GHz (Intel Corporation), 64 GB DDR4-SDRAM, and a GeForce RTX6000 ADA 48 GB graphical processing unit (Nvidia Corporation) running on a Linux system (Ubuntu 22.04.3 LTS; Canonical Ltd.) with CUDA 12.3 (Nvidia Corporation). 

**Table 1:** Depiction of the hyperparameter search for the model layers

Hyperparameter	Values tested (final selection in bold)
Initial layer, start features	10, 12, 14, 16, **20**, 24
Initial layer, stride	**1**, 2
Initial layer, maxpool stride	1, **2**, 4
Dense layers and blocks	**[6,12,24,16],** [6,12,32,32], [6,12,48,32], [6,12,64,48], [4,8,16,12]
Dense layers, *k*	12, **16**, 20, 24, 30
Dense layers, theta	**0.5**, 0.25, 0.0625
Batch size	4, **8**, 16
Epochs	12, 16, **24**, 32
Learning rate, initial value	0.00002, 0.00005, **0.0001**, 0.0005, 0.001
Learning rate, eta	0.005, **0.01**, 0.02, 0.05, 0.2, 0.5
Oversampling of positive endpoints	2, 3, 4, 8, 10

The source code of the model is available on GitHub (https://github.com/TUM-German-Heart-Center-Radiology/CNN-3D-Model).

### Statistical Analysis

To evaluate the standalone performance of the CNN model, we used a confusion matrix to analyze the pattern of prediction. In addition, several performance metrics were calculated including sensitivity, specificity, F1 score, positive and negative predictive values, precision, recall, accuracy, detection rate, and prevalence. To assess possible variability or bias, nonparametric bootstrapping was performed, using 500 resamples. The standard error of the median for model evaluation metrics was calculated and compared to the original standard error.

For assessing the value of the model in the context of conventional parameters, the prediction of the fully trained model normalized by the softmax function was used as an additional variable. The incremental value was assessed using the linear predictors of Cox multivariate models adding stepwise “extent of CAD” and the CNN model to Morise score as clinical parameter. Outcome prediction and incremental value were measured by receiver operating statistics using deLong’s test. All statistical tests were performed two-sided and a significance level of 5% was used. The statistical package R version 4.2.0 including the package rms was used for statistical analysis.

## Results

### Patient Characteristics

Figure 1 depicts the process of patient enrollment. After applying the exclusion criteria, 5562 and 714 patients were assigned to the training and test groups, respectively. An additional 4:1 random split of the training group created further subsets with 4434 patients in the training and 1128 patients in the validation subset.

The train and validation patient population consisted of 5562 patients, with a mean age of 61.1 ± 11.2 years and 66.4% of male gender. Furthermore, of the 5562 patients, 419 (7.6%) showed diabetes, 1839 (32.2%) were currently or had a history of smoking, and 1964 (34.3%) had a positive family history of cardiovascular disease. Hypercholesterolemia was found in 3125 (54.7%) and hypertension in 3112 (54.4%) patients.

The test patient population consisted of 714 patients with a median age of 61.5 ± 11.4 years and 69.3% of the cohort being of male gender. Diabetes was present in 41 (5.74%) of the patients, 263 (36.8%) had a history of or were currently smoking, and 195 (27.3%) had a positive family history of cardiovascular disease. Furthermore, hypercholesterinemia was present in 372 (52.1%) and hypertension in 317 (44.4%) patients. The Morise risk score amounted to 11.2 ± 2.76 and 10.4 ± 2.67, respectively, in the training and test population.

In the train and validation group, 760 endpoint events (175 deaths, 27 non-fatal myocardial infarction, 1 unstable angina, and 615 revascularization events) occurred during a median follow-up duration of 7.2 years. In comparison, the test cohort had a total of 83 endpoint events (6 deaths, 1 non-fatal myocardial infarction, and 77 revascularization events).

Table 2 shows a detailed overview of the characteristics of the study population.

**Table 2:** Patient characteristics

	Train and validation dataset	Test dataset	*p* Value
Number of patients	5468	714	
Age (years)	61.1 ± 11.2	61.5 ± 11.4	0.32
Male sex, *n* (%)	3794 (66.4)	495 (69.3)	0.12
Body mass index (kg/m^2^)	24.8 ± 42.1	18 ± 16.6	< 0.0001
Diabetes, *n* (%)	419 (7.6)	41 (5.74)	0.051
Smoking, *n* (%)	1839 (32.2)	263 (36.8)	0.013
Hypertension, *n* (%)	3112 (54.4)	317 (44.4)	< 0.0001
Hypercholesterolemia, *n* (%)	3125 (54.7)	372 (52.1)	0.2
CAD family history, *n* (%)	1964 (34.3)	195 (27.3)	0.00015
Morise risk score	11.2 ± 2.76	10.4 ± 2.67	< 0.0001
Extent of CAD No CAD Non-obstructive CAD (%) Obstructive CAD (%)	1143 (20.6) 3037 (54.6) 1382 (24.8)	180 (25.2) 358 (50.1) 176 (24.6)	0.016
CAD-RADS 0 1 2 3 4a 4b 5	1143 (20.6) 1128 (20.3) 1909 (34.3) 1066 (19.2) 267 (4.8) 21 (0.38) 28 (0.50)	180 (25.2) 184 (25.8) 174 (24.4) 132 (18.5) 41 (5.7) 3 (0.4) 0 (0)	< 0.0001

The follow up events are shown in Table 3.

**Table 3:** Follow-up events

	Train and validation dataset	Test dataset	*p* Value
All causes of death	175 (3.2)	6 (0.8)	< 0.001
Cardiac death	100 (1.8)	5 (0.7)	0.029
Non-cardiac death	75 (1.4)	1 (0.1)	0.002
Myocardial infarction	27 (0.5)	1 (0.1)	0.36
Unstable angina	1 (0.02)	0 (0)	> 0.99
All revascularizations	615 (11.1)	77 (10.8)	0.90
Coronary artery bypass graft surgery	34 (0.6)	3 (0.42)	0.79
Percutaneous coronary intervention	586 (10.5)	75 (10.5)	> 0.99
Composite endpoint	760 (13.7)	83 (11.6)	0.14

Data presented as absolute number (percentage)

*PCI* percutaneous coronary intervention

### Model Performance

In the validation dataset of the training process, CNN-based risk prediction for the composite endpoint had an area under the curve (AUC) of 0.823 ± 0.016. In the test dataset, AUC was 0.872 ± 0.020.

The test model’s performance was evaluated by calculating a confusion matrix (Table 4), which showed that the model was able to correctly predict 547 (including true positives and negatives) out of 714 in total evaluated cases but misclassified 167. This yielded an overall sensitivity of 80.7%, a specificity of 76.1%, and a balanced accuracy of 78.3%. The calculated positive predictive value resulted 30.7%, while the negative predictive value resulted 96.8%. The F1 score reflecting the balance between the positive predictive value and sensitivity was 44.5%. Furthermore, the model’s detection rate ranked at 9.3% and the detection prevalence at 30.5%. The actual prevalence of observed events ranked at 11.6%.

**Table 4:** Confusion matrix

Predicted negative (0)	480	16
Predicted positive (1)	151	67

A detailed overview of the performance metrics is depicted in Table 5.

**Table 5:** Performance metrics

Performance metrics	Value (%)
Sensitivity	80.1
Specificity	76.1
Balanced accuracy	78.3
Positive predictive value	30.7
Negative predictive value	96.8
F1 value	44.5
Prevalence	11.6
Detection prevalence	30.5

Key performance indicators of the CNN model

The bootstrapped standard error was 0.397, compared to the original standard error of 0.424.

Activation maps confirmed that the model detected plaques and stenoses as main source of risk calculation (Fig. 2).

Head-to-head comparison between conventional and CNN parameters is given in Table 6.

**Table 6:** A head-to-head comparison between conventional risk score and CNN parameters

	eo CAD (AUC ± SD)	CNN (AUC ± SD)	*p* Value
Test	0.891 ± 0.013	0.872 ± 0.020	0.238
Validation	0.788 ± 0.018	0.823 ± 0.016	0.059

**Fig. 4:**
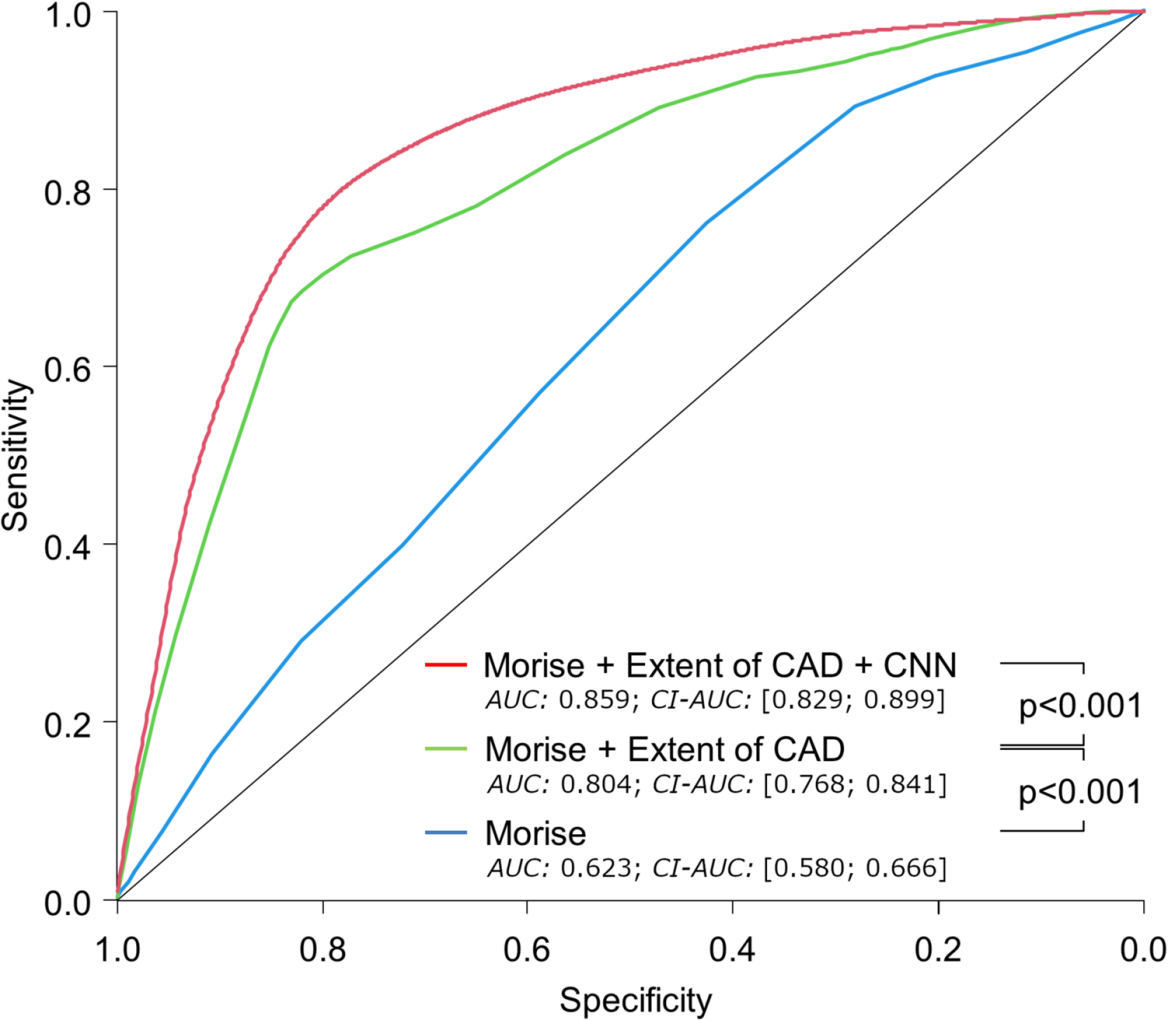
Three-step model AUC using the validation data set

In a stepwise model combining all prediction methods in the training/validation cohort, it was found that the AUC was from 0.623 ± 0.022 for Morise score alone to 0.804 ± 0.019 for adding eoCAD (*p* for difference < 0.0001) and, eventually, to 0.859 ± 0.015 by means of Morise score, eoCAD, and CNN combined (*p* for difference < 0.0001) (Fig. 4).

**Fig. 5:**
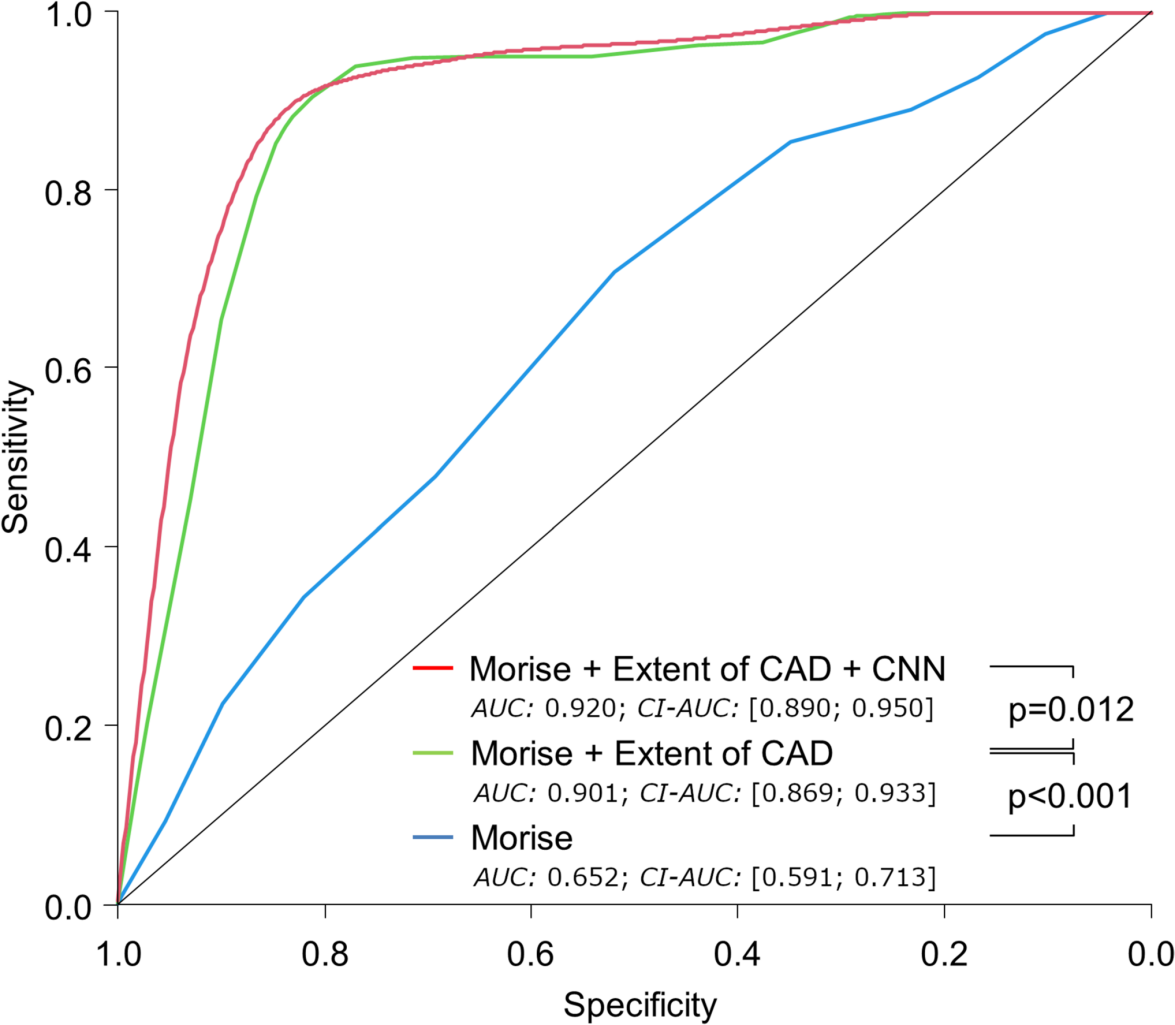
Three-step model AUC using the test data set

In the stepwise model of the test cohort, AUC increased from 0.652 ± 0.031 to 0.901 ± 0.016 (*p* for difference < 0.0001), to 0.920 ± 0.015 (*p* for difference = 0.012) (Fig. 5).

Similar results were found if using segment involvement score as conventional CT-based parameter: AUC increased from 0.652 ± 0.031 to 0.856 ± 0.019 (*p* for difference < 0.0001), to 0.889 ± 0.017 (*p* for difference = 0.0059).

## Discussion

In this study, we use 3D data, unannotated for plaque presence, originating from coronary CT angiography to train and test a CNN model for predicting major adverse cardiovascular events (MACEs) in patients with suspected coronary artery disease (CAD).

Combining CNN with CT-based parameters, primarily the extent of coronary artery disease (eoCAD), improves risk prediction for the combined endpoint over using clinical risk scores alone. This effect was determined based on area under the curve (AUC) analysis of the receiver operating characteristic (ROC) curve, showing a statistically significant improvement of the risk prediction (*p* < 0.001).

Additional metrics including a confusion matrix were calculated to provide an overview of the model’s overall performance. The model showed a robust ability in predicting outcomes, accurately identifying positive and negative cases, though it appeared to show a tendency of overpredicting the positive cases, as reflected by the detection prevalence exceeding the actual prevalence.

The model’s performance is also unlikely to be driven by random sample bias, as evidenced by the bootstrapped standard error being smaller than the original estimated one.

Furthermore, in a stepwise model, we first calculated the AUC for the Morise score, then for the Morise score combined with eoCAD, and finally for the Morise score combined with eoCAD and the CNN, demonstrating incremental improvements in risk prediction for each step both in the training and test dataset. The smaller improvement of prediction by CNN in the test model is most likely due to the better performance of the conventional CT score in a dataset exclusively from the latest scanner generation.

Being context naïve without pretraining, the model showed remarkable results. Activation maps demonstrated that it detected both plaques and stenoses, which is reassuring given the inherent black box nature of the model.

Highest activation was found on high grade stenosis as shown exemplified in Fig. 2. It remains unclear if this is caused by the high prevalence of revascularization in the composite endpoint or by a limited information content of plaque composition. Unfortunately, the low number of events prevented a training on harder endpoints.

Integrating deep learning algorithms into medical imaging analysis has gained attention because of its potential to optimize the clinical workflow, as well as allow for extraction of additional details from images [3, 6].

This study demonstrates the feasibility of using a CNN model for time-to-event analysis of a combined endpoint, which includes all-cause death, myocardial infarct, unstable angina, and all revascularization events, without requiring manual plaque annotation. To the best of our knowledge, this approach remains unique in directly using CNN on CCTA-derived images for long-term risk prediction.

Several aspects strengthen our model. First is the use of data from multiple scanner generations, which boosts generalizability across varied imaging quality. Second is the long follow-up time, which reduces label noise, captures disease progression over time, and enhances the clinical relevance of the predictions. Third, the use of large-scale data provides a solid foundation for both training and validating the model, increasing its reliability and applicability across diverse patient populations.

The model alone is not better in predicting outcome than conventional parameters and, as most other CCTA parameters, has a quite high false-positive rate. This and its inherent black box nature prevent it from being used as standalone information for clinical decision making.

However, it seems to be based on different information since prediction improves when combining it with existing information. While the activation maps show it using the plaques for risk assessment, further evaluation is needed on where the incremental value derives from.

It is interesting to note that the model still shows incremental prognostic value in the test cohort acquired on the latest scanner generation and showing a much better performance of conventional CT parameters than in the validation cohort.

Several works have explored implementing AI for analyzing cardiac imaging. In a large-scale, international multicenter study, Lin et. al. developed and trained a deep learning algorithm to quantify plaque volume and stenosis severity directly from CCTA images, using a cohort of 2757 patients. Their deep learning–based plaque volume and stenosis measurements demonstrated prognostic value for myocardial infarction after adjusting for presence of obstructive stenosis and clinical risk scores [22].

Hong et. al. focused on using deep learning for measuring lesion-specific metrics, such as minimal lumen area, percentual diameter stenosis, and contrast density difference in a smaller dataset, which consisted of 156 patients. Expert annotated 2D cross-sectional images were utilized as an input for training the CNN and the end results were compared to expert readers [23].

In another study, Masuda et al. employed a CNN to classify fibrous versus fibro-fatty plaques, using integrated backscatter intravascular ultrasound (IB-IVUS) as the reference. A cohort of 178 patients was used to evaluate the CNN’s diagnostic performance against board-certified radiologists for non-calcified coronary plaques [24].

Additionally, Ildayhid et al. developed a fully automated, unsupervised 3D deep learning model for assessing coronary artery stenosis and high-risk plaque features from CCTA scans of 1384 patients. Diagnostic accuracy of the model was evaluated against that of expert readers [3].

In comparison, our approach leverages unannotated 3D data for AI-driven risk prediction. Both the longer follow-up time and the larger dataset are two key points, which allow a more robust evaluation of the risk prediction. The 3D data allows capturing spatial relationships within the coronary arteries more effectively, potentially improving on prognostic capabilities. These key aspects highlight how our work adds to previous research.

Nonetheless, certain limitations are associated with this study. First, the data used to train the model is retrospective, which introduces potential vulnerabilities such as bias and missing or incomplete documentation. These may affect the quality of image labeling. Additionally, information collected through the follow-up conversations may be prone to recall bias.

Second, our study is based on a single-center experience. Even though a large-scale data set was used, it does not eliminate the risk of selection bias in the patient population and raises concerns about the lack in data diversity. Furthermore, the data was acquired based on institute specific scanning and post-processing methods, which could reduce the model’s generalizability. As a result, it might exhibit a decreased performance when applied to real-world data.

Third, while the model works without annotation of plaques, centerlines of vessels still need to be manually verified and adjusted. This limitation currently prohibits the model from being a fully automated application to all scans.

Fourth, the composite endpoint includes not only hard endpoints, but also revascularizations, an endpoint that is based on the individual decision of the performing physician and might be influenced by the result of the CCTA findings. To improve the value of the method for clinical decision making, further training on larger datasets with enough hard endpoints would be necessary.

## Conclusion

Our study shows the feasibility of using a CNN-based algorithm directly on CCTA images for long-term risk prediction, demonstrating significant improvement compared to traditional approaches. Further research is needed to continue exploring and optimizing the potential of this method.

## Data Availability

Data may be used in the context of a cooperation study.

## Abbreviations

2D: Two-dimensional
3D: Three-dimensional
AI: Artificial intelligence
AUC: Area under the curve
CAD: Coronary artery disease
CCTA: Coronary computed tomography angiography
CNN: Convolutional neural networks
ECG: Electrocardiogram
eoCAD: Extent of coronary artery disease
HRP: High-risk plaque
IB-IVUS: Integrated backscatter intravascular ultrasound
LAD: Left anterior descending artery
LCx: Left circumflex artery
MACE: Major adverse cardiac event
RCA: Right coronary artery
ReLU: Rectified linear unit
ROC: Receiver operating characteristic
SIS: Segment involvement score
